# Variable resistance to spinetoram in populations of *Thrips palmi* across a small area unconnected to genetic similarity

**DOI:** 10.1111/eva.12996

**Published:** 2020-05-29

**Authors:** Pan Shi, Shao‐Kun Guo, Yong‐Fu Gao, Li‐Jun Cao, Ya‐Jun Gong, Jin‐Cui Chen, Lei Yue, Hu Li, Ary Anthony Hoffmann, Shu‐Jun Wei

**Affiliations:** ^1^ Institute of Plant and Environmental Protection Beijing Academy of Agriculture and Forestry Sciences Beijing China; ^2^ Department of Entomology and MOA Key Lab of Pest Monitoring and Green Management College of Plant Protection China Agricultural University Beijing China; ^3^ School of BioSciences Bio21 Institute The University of Melbourne Melbourne VIC Australia

**Keywords:** dispersal, population genetic structure, resistance, spinetoram, *Thrips palmi*

## Abstract

The melon thrips, *Thrips palmi*, is an increasingly important pest of vegetables in northern China. Some populations have developed resistance in the field to the insecticide spinetoram. Understanding the origin and dispersal of insecticide‐resistant populations can shed light on resistance management strategies. In this study, we tested susceptibility of seven greenhouse populations of *T. palmi* to spinetoram collected from a small area of about 300 km^2^ in Shandong Province and examined population genetic structure across the area based on a segment of mitochondrial *cox1* gene and 22 microsatellite loci to infer the possible origin and dispersal of insecticide resistance. Levels of resistance to spinetoram differed among seven populations, which included one population with high resistance (LC_50_ = 759.34 mg/L), three populations with medium resistance (LC_50_ ranged from 28.69 to 34.79 mg/L), and three populations with low resistance (LC_50_ ranged from 7.61 to 8.97 mg/L). The populations were genetically differentiated into two groups unrelated to both levels of resistance and geographic distance. The molecular data indicated high levels of gene flow between populations with different levels of resistance to spinetoram and low gene flow among populations with the same level of resistance, pointing to a likely separate history of resistance evolution. Resistance levels of two tested populations to spinetoram decreased 23 and 4.6 times after five generations without any exposure to the pesticide. We therefore suspect that resistance of *T. palmi* most likely evolved in response to local applications of the insecticide. Our study suggests that the development of resistance could be avoided or resistance even reversed by reducing usage of spinetoram.

## 1 INTRODUCTION

Applications of insecticide have led to the evolution of resistance in many insect pests (Nauen, Slater, Sparks, Elbert, & Mccaffery, 2019). Insecticide resistance management (IRM) has become one of the components of pest control practices aimed at extending the useful life of a chemical against a pest (Brattsten, Holyoke, Leeper, & Raffa, 1986; Roush & Tab ashnik, 2012). Insecticide resistance can evolve and spread out from a single original population or independently evolve in multiple populations (Andreev, Kreitman, Phillips, & Beeman, 1999; Daborn et al., 2002; Shi et al., 2019). Understanding the origin and dispersal of insecticide resistance especially in its early stage can provide information for identifying resistance mechanisms and managing further resistance evolution (Daborn & Le Goff, 2004; Hawkins, Bass, Dixon, & Neve, 2018).

Tracing the origin and spread of resistance can be challenging. When resistance alleles spread out from a single origin via human activities, populations with pesticide resistance are often geographically and/or genetically connected through gene flow (Daborn et al., 2002; Raymond, Callaghan, Fort, & Pasteur, 1991). In cases where resistance has independent origins, developing in geographically distant populations, some populations may remain susceptible to an insecticide while others have varying levels of resistance, and these resistance patterns may be unconnected to geographic proximity (Shi et al., 2019). This pattern can be produced by population differences in local selection intensity. Population genetic approaches provide a useful approach to testing these scenarios, because they can trace the dispersal of individuals and possible spread and selection for insecticide resistance and also test how this spread coincides with resistance (Crossley, Chen, Groves, & Schoville, 2017; Fu, Epstein, et al., 2017; Pélissié, Crossley, Cohen, & Schoville, 2018; Shi et al., 2019; Yang et al., 2019).

Here, we compare molecular differentiation to resistance patterns in the melon thrips, *Thrips palmi* Karny (Insecta: Thysanoptera: Thripidae). This species is an economically important agricultural pest on vegetables. It causes severe injury to infested crops by ovulating, feeding directly, and transmitting plant virus from *Orthotospoviruses* (Rotenberg, Jacobson, Schneweis, & Whitfield, 2015; Stuart, Gao, & Lei, 2011). Originating from tropical countries of Asia, *T. palmi* was introduced and became established across South‐East Asia, South America, the Caribbean, Florida, Australia, and West Africa (Cannon, Matthews, & Collins, 2007). In recent years, this species spread to northern China and became a serious pest in greenhouse vegetables (Cao et al., 2019). Unlike the invasive western flower thrips, *Frankliniella occidentalis*, which rapidly spread into most areas of China after initial reports, likely accelerated by human activities (Cao et al., 2017), *T. palmi* expanded its range of distribution in a pattern that fits a stepping stone model, forming genetic structure among geographically distinct populations (Cao et al., 2019).

Management of *T. palmi* has been heavily reliant on chemical control (Bao et al., 2014). However, the species has a high capability of developing resistance to numerous pesticides (Bao et al., 2014; Bao & Sonoda, 2012). Spinetoram is currently one of the remaining insecticides available to control thrips around the world (Cannon et al., 2007; Mouden, Sarmiento, Klinkhamer, & Leiss, 2017; Reitz et al., 2019). Resistance to spinetoram has developed in populations of *T. palmi* in Japan (Bao et al., 2014), but this insecticide continues to be widely used in controlling populations in China. However, a recent study showed that some populations of *T. palmi* have developed varying levels of resistance to spinetoram in northern China, although most populations remain susceptible (Gao, Gong, Cao, et al., 2019). For example, the resistance of *T. palmi* to spinetoram is variable in the Beijing area of China, with LC_50_ values among populations ranging from 1.69 to 19.69 mg/L (Gao, Gong, Cao, et al., 2019). It is still not clear whether the resistance of *T. palmi* to spinetoram has evolved in response to local selection pressures or whether it is dictated by gene flow. Understanding the development and spread of resistance in *T. palmi* to spinetoram will help to develop strategies for resistance management.

In this study, we investigated the development and spread of resistance of *T. palmi* to spinetoram in a small region of Shandong Province by comparing resistance levels with genetic structure among populations and changes in resistance after rearing thrips without exposure to pesticides. Our study was based on the following conjectures: When populations with similar resistance share a similar genetic background, we considered them as forming one cluster and having a single origin of resistance; on the other hand, when there are multiple populations with resistance and these are not connected genetically, we suspect that resistance has multiple origins. Based on our knowledge of the biology and resistance status of *T. palmi*, we hypothesized that resistance was more likely to develop independently in multiple populations rather than being solely a consequence of gene flow.

## 2 MATERIALS AND METHODS

### 2.1 Samples

**Figure 1:**
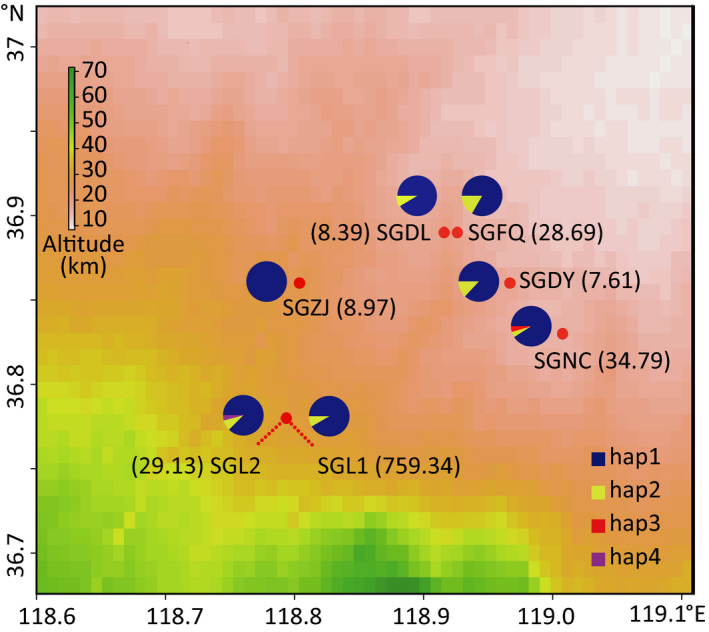
Collection map of seven populations of *Thrips palmi* (red points) collected from Shouguang, Shandong Province, their 50% lethal concentrations (LC_50_, number in brackets, mg/L) and proportion of mitochondrial haplotypes in each population (pie charts). All populations were collected from different greenhouses

To compare spinetoram resistance and genetic structure in populations of *T. palmi*, we sampled seven populations from greenhouses in six villages, involving two collections from eggplant, three from pepper, and two from cucumber (Table 1). These populations were collected from Shouguang in Shandong Province, where is a large area for vegetable production and where the control of *T. palmi* has been heavily reliant on spinetoram in the past few years. Due to the reduced control efficacy of spinetoram, the frequency of application of this pesticide has been reduced to 1–3 times per growing season in recent years. All samples were collected on November 20, 2018, except for SGL1, which was collected on July 11, 2018. Samples were collected from a core planting area covering about 300 km^2^ (Figure 1, Table S1). A set of around 3,000 thrips collected from one host plant crop in a greenhouse was considered to represent a population. These thrips were collected from at least 24 sites scattered across the greenhouse crop. The populations were kept in net bags and taken to the laboratory for bioassays and immediate genotyping. For each population, both randomly selected male and female adults were used for bioassays to estimate their susceptibility to spinetoram. Due to haplodiploidy in thrips (haploids develop into males and diploids into females) (Moritz, 1997), we used 24 diploid adult females per population for genetic analyses so that heterozygosity could be computed. One individual was randomly selected from each of the 24 collection sites in a crop for genotyping to decrease the likelihood of close relatives being included in the population sample.

**Table 1:** Host plant, pesticide usage status, and resistance level to spinetoram for populations of *Thrips palmi*

Population	Host plant	Spi. dosage (mg/L)	Frequency (spi./all)	Resistance level	LC_95_ (95% CI) (mg/L)	LC_50_ (95% CI) (mg/L)
SGDY	Cucumber	60	2/4	Low	208.34 (111.06–624.73)	7.61 (4.57 ~ 10.82)
SGDL	Cucumber	120	2/5	Low	169.68 (118.91–271.13)	8.39 (6.25–10.70)
SGZJ	Hot pepper	60	3/6	Low	282.84 (169.74–573.54)	8.97 (6.41–12.00)
SGFQ	Sweet pepper	120	2/8	Medium	793.21 (414.31–3056.75)	28.69 (9.34–50.59)
SGL2	Eggplant	120	3/8	Medium	2,797.77 (970.04–14,923.55)	29.13 (19.93–46.34)
SGNC	Sweet pepper	180	2/10	Medium	5,540.48 (1821.71–37052.21)	34.79 (20.50–55.97)
SGL1	Eggplant	120	2/12	High	15,587.02 (4,860.33–114,634.77)	759.34 (433.74–1916.57)
SGL1‐F5^a^	Cucumber	NA	NA	Medium	162.31 (125.29–234.83)	33.12 (29.47–43.02)
BJFS^b^	Cucumber	90	2/2	Low	23.93 (16.44–38.67)	1.69 (1.33–2.14)
BJFS‐F5^a^	Cucumber	NA	NA	Low	52.74 (35.08–90.98)	0.37 (0.19–0.56)

Note

The first seven populations collected from Shouguang of Shandong Province were used for population genetics analysis.

Abbreviations: CI, confidence intervals; NA, not available; Spi., spinetoram.

^a^ SGL1‐F5 and BJFS‐F5 are the SGL1 and BJFS populations after rearing in the laboratory without exposure to pesticides for five generations. History of pesticide usage, including dosage of spinetoram per application and spray frequency of spinetoram and all insecticides per growing season, was obtained from farmer records.

^b^ From Gao, Gong, Cao, et al., 2019.

Additionally, we used another population on cucumber in July 2018 from the Beijing area (BJFS) (Gao, Gong, Cao, et al., 2019) , about 400 km away from Shouguang, where the level of resistance was expected to be low compared to samples collected from Shouguang due to the low usage of spinetoram in the Beijing area. This population and one population collected from Shouguang (SGL1) as described above were reared in the laboratory for five generations without pesticide exposure on cucumber to examine changes in spinetoram susceptibility across generations (Table 1).

### 2.2 Bioassay

Spinetoram 6% SC (Dow AgroSciences Company) was used in a leaf‐dipping bioassay for testing susceptibility of *T. palmi* (Wang et al., 2016). The original concentrations of spinetoram were determined based on pretests and then were serially diluted into eight concentrations using distilled water containing 0.1% Triton X‐100 (Beijing Solar BioScience and Technology Limited Company). Three duplicates were set for each concentration of spinetoram. All leaves used for bioassays were grown in a greenhouse without exposure to insecticides, and leaves came from the same plant type as where the species was collected. The leaves were cut to fit plastic containers and dipped in spinetoram solutions for ten seconds before air drying at room temperature. We used 0.2% agar in the bottom of the containers to avoid the leaves drying out. In total, 20–25 adults *T. palmi* were transferred to leaves and then the containers were covered with gauze (200 μm × 200 μm). The treated *T. palmi* were kept at 25°C, 40%–60% relative humidity, and a photoperiod of 16L: 8D. Mortality was recorded after 48 hr. Individuals unable to move were considered as dead. Control leaves were treated with 0.1% Triton X‐100 solution. All control moralities were below 10% in the bioassays. The lethal concentrations of 50% (LC_50_) and 95% (LC_95_) and other parameters were estimated through DPS software (Tang & Zhang, 2013).

### 2.3 Genotyping

Total DNA was extracted from individual specimens using DNeasy Blood and Tissue Kit (Qiagen). For nuclear markers, we used 22 microsatellite loci developed in a previous study (Gao, Gong, Ma, et al., 2019). A fluorescence‐labeled PC‐tail (5′ CAGGACCAGGCTACCGTG 3′) was used to label the PCR products (Blacket, Robin, Good, Lee, & Miller, 2012; Schuelke, 2000). Conditions for PCR amplification were described in Gao, Gong, Ma, et al. (2019). The size of amplified PCR products was determined using an ABI 3730xl DNA Analyzer with GeneScan 500 LIZ size standards. Alleles were identified with GENEMAPPER version 4.0 (Applied Biosystems, USA).

For the mitochondrial gene, a segment of cytochrome c oxidase subunit I (*cox1*) was amplified with primer pairs TP‐AF (5′ TTTCGTCTAACCATAAAGATATCGG 3′) and TP‐AR (5′TAAACTTCTGGGTGCCCAAAAAATCA 3′) (Cao et al., 2019). Polymerase chain reaction (PCR) was conducted with the following program: an initial denaturation for 3 min at 94°C, followed by 35 cycles of 30 s at 94°C, 15 s at 52°C and 1 min at 68°C, and a subsequent final extension for 10 min at 68°C. Amplified products were purified and sequenced directly from both strands using an ABI 3730xl DNA Analyzer by Tsingke Biotechnology Co. Ltd.

### 2.4 Genetic diversity analysis

For microsatellite loci, statistics measuring genetic diversity, including allele frequencies, allele numbers (A_T_), observed heterozygosity (Ho), and expected heterozygosity (He) were estimated by the macros in Microsatellite Tools. Tests of Hardy–Weinberg equilibrium (HWE) at each locus, as well as estimation of pairwise population differentiation (*F*_ST_) (Weir & Cockerham, 1984) and inbreeding coefficients (F_IS_) were undertaken with GENEPOP version 4.2.1 (Rousset, 2008). Allele richness (A_R_) was calculated by FSTAT V2.9.3 (Goudet, 1995).

For mitochondrial DNA, sequences of the *cox1* were aligned with CLUSTALW (Larkin et al., 2002) implemented in MEGA version 7.0 (Kumar, Stecher, & Tamura, 2016). Number of polymorphic sites (S), haplotype diversity (h), and nucleotide diversity (π) were estimated in DnaSP version 6.0 (Rozas et al., 2017).

### 2.5 Population genetic analyses

To examine genetic structure across the populations, phylogenetic relationships among the populations were inferred with POPTREE2 (Takezaki, Nei, & Tamura, 2009) using the neighbor‐joining (NJ) method based on microsatellite loci. In addition, Bayesian model‐based clustering implemented in STRUCTURE version 2.3.4 (Pritchard, Stephens, & Donnelly, 2000) and a discriminant analysis of principal components (DAPC) using R package adegenet version 2.0.1 (Jombart, 2008) were performed on variation at the microsatellite loci to assess population genetic structure.

A Mantel test was used to evaluate the correlation between pairwise genetic differentiation (*F*_ST_) and geographic distance. A Mantel test was also used to compare genetic differentiation to population variation in insecticide susceptibility. Tests were run in the R package *ade4* (Jensen, Bohonak, & Kelley, 2005). The population difference in susceptibility was calculated as the difference of LC_50_ between population pairs divided by the highest LC_50_ value.

Migration rates within recent generations among populations of *T. palmi* were calculated with BAYESASS version 3.0.4 (Gregory, 2003) based on microsatellite loci. The convergence of analysis was checked in TRACER 1.7 (Rambaut, Drummond, Xie, Baele, & Suchard, 2018).

## 3 RESULTS

### 3.1 Susceptibility of *T. palmi* to spinetoram in seven greenhouse populations

LC_50_ values of seven greenhouse populations of *T. palmi* collected from Shouguang varied from 7.61 to 759.34 mg/L, while LC_95_ values varied from 169.68 to 15,587.02 mg/L, showing a high level of variation in susceptibility to spinetoram among populations across the small area sampled (Table 1). Compared with the recommended dosage of 45 mg/L to 75 mg/L for field control of thrips, the seven populations from Shouguang showed resistance to spinetoram. Resistance in the populations was 63 to 6,328 times higher compared with a population of *T. palmi* from Hainan Province with an LC_50_ value of 0.12 mg/L (Gao, Gong, Cao, et al., 2019), while resistance was 2.2 to 223 times higher compared with a strain from Japan with an LC_50_ of 3.4 mg/L (Bao et al., 2014). The seven populations were classified into three levels of resistance to spinetoram: One population (SGL1) showed a high level of resistance with LC_50_ of 759.34 mg/L, three populations (SGFQ, SGL2, SGNC) showed medium levels of resistance with LC_50_s ranging from 28.69 mg/L to 34.79 mg/L, while three populations (SGDY, SGDL, SGZJ) showed relatively low resistance with LC_50_ values ranging from 7.61 mg/L to 8.97 mg/L.

### 3.2 Genetic diversity of *T. palmi* in seven greenhouse populations

For the mitochondrial *cox1* gene, four haplotypes were found, and nucleotides diversity was low, ranging from 0 to 0.0004. Hap1 was the most common haplotypes followed by Hap2, and these two haplotypes were found in all populations except for SGZJ that only had Hap1. Additionally, there was one Hap3 individual and one Hap4 individual in the populations SGNC and SGL2, respectively (Figure 1, Table 2). Proportions of the four *cox1* haplotypes did not differ significantly across the seven populations (Figure 1, Table 2).

**Table 2:** Genetic diversity parameters in populations of *Thrips palmi* based on mitochondrial *cox1* and 22 microsatellite loci

Population	Mitochondrial DNA		Microsatellite loci				
	H	π	A_R_	A_T_	H_e_	H_o_	F_IS_
SGDY	2	0.00041	4.1818	92	0.4490	0.4280	0.0477
SGDL	2	0.00061	4.4545	98	0.4664	0.4527	0.0300
SGZJ	1	0.00057	4.8636	107	0.5684	0.5284	0.0718
SGFQ	2	0.00072	4.5909	101	0.5140	0.5152	−0.0022
SGL2	3	0.00000	4.1364	91	0.4749	0.4811	−0.0134
SGNC	3	0.00045	4.5455	100	0.4930	0.4451	0.0992
SGL1	2	0.00040	4.8636	107	0.5657	0.5701	−0.0079
SGL1‐F5	2	0.00116	4.4091	97	0.5509	0.5170	0.0627
BJFS	2	0.00089	4.5000	99	0.5609	0.5473	0.0247
BJFS‐F5	2	0.00057	4.4751	99	0.5645	0.5615	0.0052

Abbreviations:

H, number of haplotypes; π, nucleotide diversity; A_R_, allelic richness; A_T_, total number of alleles; H_e_, expected heterozygosity; H_o_, observed heterozygosity; F_IS_, inbreeding coefficient.

For the microsatellite loci, we found 696 alleles among the 168 females of *T. palmi* characterized for the 22 microsatellite loci. The average allelic richness (A_R_) varied from 4.14 to 4.86. The observed heterozygosity (Ho) tended to be lower‐than‐expected heterozygosity (He) and the inbreeding coefficients (F_IS_) was low (−0.01–0.10) (Table 2). Nine out of 154 population‐locus pairs showed deviation from HWE (*p* < .05); however, none of the loci showed deviation in all populations, and no population showed HWE deviation at all loci (Table S2).

### 3.3 Population genetic structure of seven greenhouse populations

**Figure 2:**
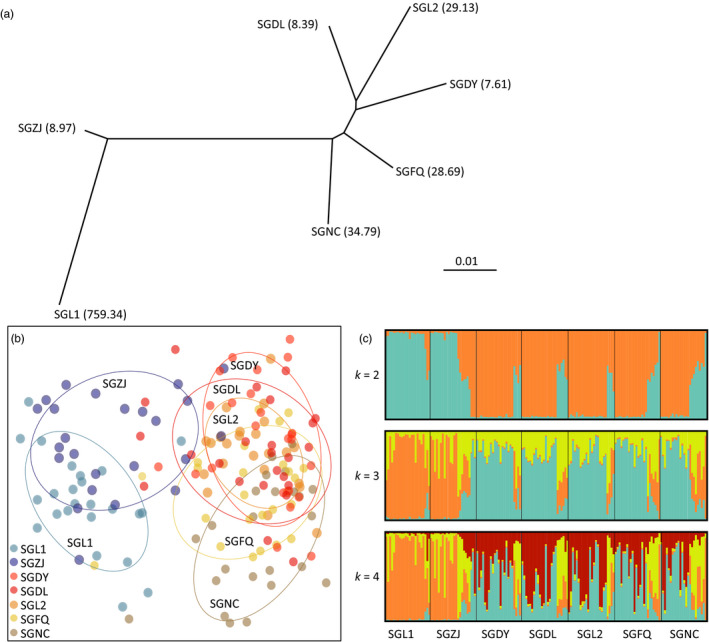
Population genetic structure of seven greenhouse populations of *Thrips palmi* collected from Shouguang, Shandong Province based on 22 microsatellite loci. (a) Phylogenetic relationships among the seven populations inferred by POPTREE. Number in brackets shows LC_50_ value of each population; (b) scatter plot of DAPC analysis of seven populations. Individuals from the same population are indicated by the same colored points; (c) Clusters of all individuals when k is 2, 3, and 4 inferred from STRUCTURE analysis. The optimal k was 2 determined based on delta K. Each cluster is indicated by one color. Each vertical line indicates an individual. The proportion of a cluster's genotype present in one individual is represented by the length of the corresponding color bar

For microsatellite loci, pairwise *F*_ST_ values among seven populations ranged from 0.0005 to 0.1258 (Table 3). A relatively low level of differentiation was found among most population pairs (0.0005 < *F*_ST_ <0.05), but a relatively higher level of differentiation was found between SGZJ or SGL1 and the other populations (0.05 < *F*_ST_ <0.1258). The highest level of differentiation was found between SDL1 and SGNC (*F*_ST_ = 0.1258, *p* < .01). Both POPTREE and DAPC analysis indicated that all populations grouped into two clusters (Figure 2a,b). STRUCTURE analysis revealed similar genetic differentiation patterns among populations (Figure 2c).

**Table 3:** Pairwise population differentiation (*F*_ST_) among seven greenhouse populations of *Thrips palmi* collected from Shouguang in Shandong Province estimated from 22 microsatellite loci

Population	SGDY	SGDL	SGZJ	SGFQ	SGL2	SGNC
SGDL	0.0038					
SGZJ	0.0716**	0.0590**				
SGFQ	0.0039	0.0059	0.0527**			
SGL2	0.0054	0.0005	0.0634**	0.0026		
SGNC	0.0172**	0.0102**	0.0575**	0.0010	0.0093**	
SGL1	0.1258**	0.1111**	0.0095	0.0968**	0.1133**	0.0892**

* *p* < .05;

** *p* < .01 after 1,000 bootstraps.

### 3.4 Correlation between genetic differentiation and geographic distance or resistance difference

**Figure 3:**
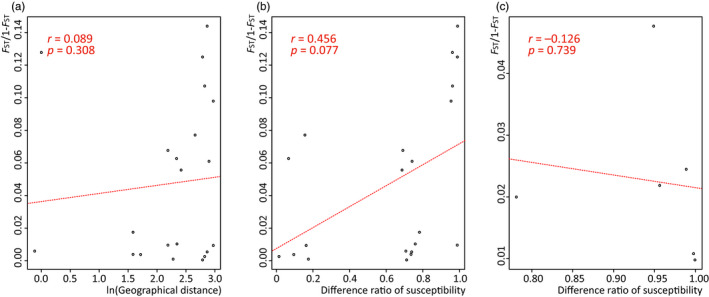
Correlations between population genetic differentiation and geographic distances (a) and resistance to spinetoram (LC_50_) (b) among seven greenhouse populations from Shandong Province and between greenhouse populations and laboratory populations (c) analyzed by Mantel tests. *r*, correlation coefficient; *p*, *p* value. Ratio of resistance of each population pair was calculated from 50% lethal concentration (LC_50_)

Mantel tests indicated that there was no correlation between genetic distance and geographic distance (*r* = .089, *p* = .308, Figure 3a) or susceptibility to spinetoram (*r* = .456, *p* = .077, Figure 3b). The highest genetic differences were found between SGL1 with low susceptibility and the other populations, followed by differences between SGZJ and the other populations. Some populations with a similar level of susceptibility were genetically distant, such as SGZJ and SGDY (*F*_ST_ = 0.0716, *p* < .01), and SGZJ and SGDL (*F*_ST_ = 0.0590, *p* < .01); and some populations with different levels of susceptibility showed low genetic differentiation, such as SGDL and SGL2 (*F*_ST_ = 0.0005, *p* < .01), and SGDY and SGL2 (*F*_ST_ = 0.0054, *p* = .1917).

### 3.5 Gene flow among seven greenhouse populations

**Figure 4:**
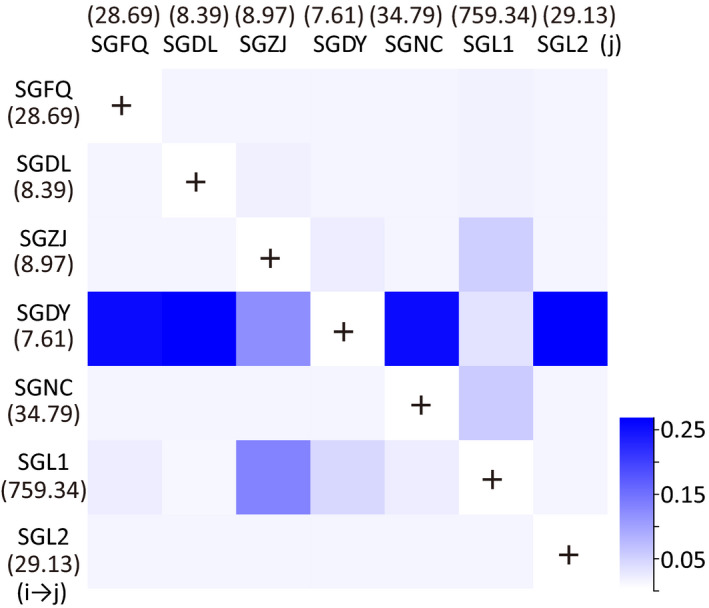
Heatmap of gene flow among seven greenhouse populations of *Thrips palmi* collected from Shouguang, Shandong Province, based on microsatellites estimated using BAYESASS. Dark color indicates high levels of gene flow, while the light color indicates low levels of gene flow

A relatively high level of contemporary gene flow was found between four populations with varied levels of resistance to population SGDY (m ranged from 0.2575 to 0.2671) and between SGZJ and SGL1 which had a large difference in resistance to spinetoram (m = 0.1291 from SGZJ to SGL1, m = 0.0523 in the reverse direction) (Figure 4). Low levels of gene flow were found among the three populations with a medium level of resistance to spinetoram.

### 3.6 Variation of susceptibility and genetic structure in the absence of spinetoram

After rearing the two populations in the laboratory for five generations without exposure to the pesticide, the resistance level of *T. palmi* decreased 23‐fold (LC_50_ decreased from 759.34 to 33.12 mg/L) and 4.6‐fold (LC_50_ decreased from 1.69 to 0.37 mg/L), respectively, compared with the SGL1 and BJFS source populations (Table 1) (Gao, Gong, Cao, et al., 2019) .

Genetic diversity estimated from microsatellites did not change significantly after five generations. The F_IS_ increased from −0.0079 to 0.0627 in SGL1 and decreased from 0.0247 to 0.0052 in BJFS (Table 2). For the mitochondrial *cox1* gene, the type of haplotype (Hap 1 and Hap 2) did not change in both populations after five generations, while the nucleotide diversity increased in SGL1 and decreased in BJFS (Table 2).

**Figure 5:**
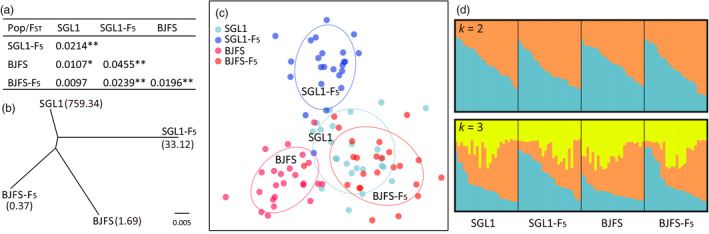
Population genetic differentiation among two greenhouse populations and their laboratory‐derived populations based on 22 microsatellite loci. (a) Pairwise population differentiation was calculated in GenePop (*p < .05; **p < .01); (b) phylogenetic relationships among populations inferred by POPTREE. Number in brackets show LC_50_ value of each population; (c) scatter plot of DAPC analysis of population genetic structure. Individuals from the same population are indicated by the same colored points; (d) clusters of all individuals when k is 2 and 3 inferred from STRUCTURE analysis. The optimal k was 1 based on delta K

Pairwise *F*_ST_ analyses showed both populations diverged significantly from their source populations after five generations, with pairwise *F*_ST_ values of 0.0214 (*p* < .01) for SGL1 and SGL1‐F5, and 0.0196 (*p* < .01) for BJFS and BJFS‐F5 (Figure 5a). The SGL1‐F5 population showed the highest differentiation with both its source population and the BJFS and BJFS‐F5 populations. The SGL1 population was not significantly differentiated from BJFS‐F5 (*F*_ST_ = 0.0097, *p* = .0874). Population genetic structure analysis using POPTREE showed that the laboratory population and its source population grouped together (Figure 5b), while DAPC and STRUCTURE did not reveal a clear pattern of genetic structure (Figure 5c,d).

## 4 DISCUSSION

### 4.1 Development of spinetoram resistance in populations of *T. palmi*

We found varying levels of susceptibility to spinetoram among the seven populations of *T. palmi* collected from a relatively small area of Shouguang, Shandong Province. Compared with a susceptible population of *T. palmi* from Hainan Province (LC_50_ = 0.12 mg/L) (Gao, Gong, Cao, et al., 2019), a strain from Japan (LC_50_ = 3.4 mg/L) (Bao et al., 2014), and the baseline susceptibility of *F. occidentalis* to spinosad (LC_50_ = 0.594 mg/L) (Eger, Stavisky, & Funderburk, 1998), the seven populations of *T. palmi* had varied levels of spinetoram resistance. The difference between the highest (SGL1 with LC_50_ of 759.34) and the lowest (SGDY with LC_50_ of 7.61) resistance levels in populations is over 99‐fold. These resistance levels would likely have affected control of the thrips, given that recommended concentrations for thrips control are set at 45 mg/L to 75 mg/L. Spinetoram resistance has been reported in many pests including the western flower thrips, *Frankliniella occidentalis,* as well as in *T. palmi* (Bao et al., 2014; Espinosa, Bielza, Contreras, & Lacasa, 2002; Gao, Gong, Cao, et al., 2019; Wan et al., 2018; Wang et al., 2016). Our data suggest that resistance of *T. palmi* to spinetoram will be an increasing problem in China (Gao, Gong, Cao, et al., 2019) with an ongoing selection expected in populations where the LC_50_ remains below field rates.

We also found that resistance levels of *T. palmi* declined after five generations without insecticide exposure. Due to the need for large numbers of individuals when undertaking bioassays, we were unable to examine the susceptibility of each generation reared successively. This made it impossible to clearly separate environment‐induced resistance from genetically controlled resistance. However, with the source and laboratory populations coming from the same hosts, we suspect that genetic changes are involved in the reduction of resistance, reflecting fitness costs associated with resistance alleles. We also found significant genetic differentiation between the F5 generation and the source generation, which was unrelated to resistance. These may occur as a consequence of genetic drift in the lines or rapid genetic changes as a consequence of adaptation to laboratory conditions (Hoffmann & Ross, 2018), assuming that some microsatellite loci are linked to loci under laboratory selection. A decrease in spinetoram resistance in the absence of ongoing selection for resistance was previously described in *Thrips hawaiiensis* (Fu et al., 2018). Fitness costs of spinetoram resistance have also previously been suggested for *F. occidentalis* and *T. hawaiiensis* (Fu, Li, et al., 2017; Li et al., 2017). Current variation in resistant levels to spinetoram in greenhouse populations of *T. palmi* might partly reflect these costs balanced against ongoing selection for resistance.

### 4.2 Contribution of gene flow to resistance in greenhouse populations of *T. palmi*

In a genetic analysis of *T. palmi* across a larger area than in the current study but including the area from which the current populations were sourced, populations from northern China formed a genetic cluster (Cao et al., 2019). This is consistent with our analysis of the mitochondrial *cox1* gene, which showed that all individuals were dominated by one haplotype. Nevertheless, we have found substantial substructuring within this northern cluster based on microsatellite markers. If resistance is associated mainly with gene flow, we might therefore still expect an association between genetic distance and resistance as assessed through the bioassays. However, there was no correlation between the level of resistance in populations and their overall genetic similarity as assessed by microsatellites. In both the genetic clusters we identified here, populations with varying levels of resistance were found, and within the smaller genetic cluster, the SGL1 population with a high‐level resistance and the SGZJ population with low resistance were closely related. Within the larger cluster, five populations with both low and medium levels of resistance were closely genetically related. Although gene flow may lead to a low level of differentiation among populations after introduction, our analysis indicated no association between resistance levels and either genetic differentiation or genetic exchange.

The lack of association between genetic distance as assessed by microsatellite markers and resistance levels may provide information on the origin of resistance. When resistance first arises in a population and spreads to other populations, there may be disequilibrium between microsatellite alleles and resistance alleles that persists for a short time depending on the location of the microsatellites relative to resistance loci and levels of recombination. However, this depends on microsatellite markers being on the same chromosome as resistance alleles. As recombination occurs across generations, any association between microsatellite alleles and resistance is expected to break down rapidly, although linkage disequilibrium may be maintained for many generations in polymorphisms near selected resistance alleles (Daborn et al., 2002). Tight linkage is unlikely for the few microsatellite loci scored here, whereas markers in linkage disequilibrium are much more likely to be discovered when thousands of SNP markers are scored across the genome (Endersby‐Harshman et al., 2019).

In the present case, the lack of a correlation between resistance and genetic distance may reflect a difference in the intensity of selection for resistance in a population on resistance mutations found throughout the geographic range of the species and/or a separate geographic origin for resistance alleles. The latter seems less likely since our study showed that all tested populations exposed to spinetoram had developed at least some resistance to spinetoram compared to previously reported levels of susceptibility (Bao et al., 2014; Gao, Gong, Cao, et al., 2019). Therefore, there may have been a single origin of resistance alleles, with local selection pressures driving them to different frequencies in populations within the area we examined. Further studies on spinetoram resistance mechanisms in these *T. palmi* populations and patterns of polymorphism around the selected alleles involved will help to resolve the role of local selection versus independent origin.

### 4.3 Potential factors influencing spinetoram resistance in *T. palmi*

The development of insecticide resistance can be influenced by many factors and resistance itself can have a complex or simple genetic basis (Crossley et al., 2017). The resistance of spinetoram in insect pests may be associated with target‐site mutation, enhanced detoxification and changes in gene transcription (Bao et al., 2014; Baxter et al., 2010; Berger et al., 2016; Somers, Nguyen, Lumb, Batterham, & Perry, 2015; Wan et al., 2018; Wang et al., 2016). For *T. palmi*, resistance to spinetoram can be conferred by the G275E mutation in the target nicotinic acetylcholine receptor α6 subunit and cytochrome P450‐mediated detoxification as identified in Japanese populations (Bao et al., 2014). However, the resistance mechanism of *T. palmi* in our tested populations (and whether it has a simple or polygenetic basis) is not known.

Insecticide resistance can be influenced by environmental factors, such as temperature and host plant (Dermauw, Pym, Bass, Van Leeuwen, & Feyereisen, 2018) and transgenerational effects of insecticides (Brevik, Lindstrom, McKay, & Chen, 2018). *T. palmi* is one of the main pests on greenhouse vegetables in the Shouguang area. The greenhouses provide suitable conditions for the continuous presence of thrips throughout the year. To control this pest, farmers need to spray pesticides intensively, which poses a high selective pressure and leads to insecticide resistance of this species.

In our study, we used plant species from which the thrips were collected for bioassays to avoid any change of susceptibility due to the host shift. When we consider the resistance of *T. palmi* to spinetoram in the tested population from the perspective of the host plant, we note that the resistance of populations collected on pepper and eggplant is higher than that from cucumber (Table 1). This might indicate that some host plants increase the resistance of *T. palmi* to spinetoram (Dermauw et al., 2018). Additionally, different levels of resistance on host plants may arise from the frequency of insecticide application on different host plants. In the field, feeding by a relatively low density of *T. palmi* can lead to significant damage of eggplant and sweet pepper, reducing the price of these vegetables, whereas on cucumber damage levels are less. For a given pest population density, more applications of pesticides might, therefore, be expected on eggplant and sweet pepper than on cucumber, generating more intense selection.

Development of spinetoram resistance may be accelerated by the haplodiploid mode of reproduction of *T. palmi* (Bielza, Quinto, Fernandez, Gravalos, & Contreras, 2007). The evolutionary dynamics of genes in haplodiploids share many features with X‐linked genes and are different from diploid (autosomal) genes in many respects (Hedrick & Parker, 1997). A simulation study showed that resistance develops at a faster rate under haplodiploid reproduction than under diploid reproduction when a resistance allele is recessive, and at a similar rate when a resistance allele is dominant or semi‐dominant (Denholm, Cahill, Dennehy, & Horowitz, 1998). Many studies have found that spinosyn resistance is recessive in insects (Bielza et al., 2007; Wang et al., 2019). Recessive inheritance and haplodiploidy may contribute to the development of resistance in *T. palmi* to spinetoram (Bielza et al., 2007). However, males are generally less tolerant to pesticides than females in both haplodiploid and diploid arthropods. Simulations considering between‐sex differences in insecticide tolerance show that resistance evolution can then be slower in haplodiploids than in diploids (Carrière, 2003). In our study, we randomly selected adults from each population irrespective of sex to represent average resistance levels of natural populations, and further studies on sex‐specific differences in resistance are needed to understand the influence of haplodiploidy on development of insecticide resistance in *T. palmi*.

### 4.4 Implications for pest management

Field control of thrips is heavily reliant on spinetoram globally (Cannon et al., 2007; Mouden et al., 2017; Reitz et al., 2019). It is time to develop management strategies for *T. palmi* targeting this pesticide before widespread resistance develops. Our study found that resistance to spinetoram developed to different levels in a small area, suggesting that insecticide pressures contribute to resistance levels. The varied level of resistance suggests that spinetoram resistance alleles in *T. palmi* are not fixed in all populations. Although resistance can persist over many generations in the absence of pesticide selection in *F. occidentalis* (Bielza et al., 2008; Brødsgaard, 1994), we found that resistance to spinetoram rapidly declined in *T. palmi* in the absence of the pesticide. This suggests that it may be possible to avoid further development of resistance or even restore susceptibility by reducing pesticide applications. We also found high levels of gene flow between some populations likely mediated by seedling transport; thus, dispersal of *T. palmi* from resistant to susceptible populations should be avoided.

## 5 CONCLUSION

We found varying levels of spinetoram resistance among populations collected from a small area. Spinetoram resistance was unrelated to genetic distance, indicating that resistance of *T. palmi* most likely evolved in response to local applications of the insecticide, as further highlighted by independent changes in susceptibility to spinetoram and genetic differentiation after thrips were reared in the laboratory without insecticide. The results provide information on the regional management of insecticide resistance and the possibility of recovering susceptibility in this pest before resistance alleles become fixed across China (Gao, Gong, Cao, et al., 2019). Our study indicates how the incorporation of population genetic approaches into insecticide resistance research can help to elucidate patterns of resistance development in the field and inform insecticide resistance management.

## CONFLICT OF INTEREST

None declared.

## AUTHOR CONTRIBUTIONS

Shu‐Jun Wei conceived and designed the study; Pan Shi, Shao‐Kun Guo, Yong‐Fu Gao, Ya‐Jun Gong and Jin‐Cui Chen conducted the field and bioassay works; Pan Shi and Yong‐Fu Gao conducted molecular works; Pan Shi, Li‐Jun Cao, Yong‐Fu Gao and Shu‐Jun Wei analyzed the data; Shu‐Jun Wei, Ary Hoffmann, Lei Yue and Hu Li discussed the results; Pan Shi, Shu‐Jun Wei and Ary Hoffmann wrote the manuscript.

## DATA ARCHIVING STATEMENT

Microsatellite data and mitochondrial *cox1* gene sequences used in the study were deposited in Dryad repository: https://doi.org/10.5061/dryad.bnzs7h476.

## Supporting information

Table S1‐S3
