# Supplementary material for: Variable resistance to spinetoram in populations of Thrips palmi across a small area unconnected to genetic similarity
Source: Evol Appl. 2020 May 29;13(9):2234–45. doi: 10.1111/eva.12996 (PMC7513702; doi:10.1111/eva.12996)
Supplement: Supplementary file 1 — Table S1‐S3 [file EVA-13-2234-s001.docx]

**Table S1** Information on *Thrips palmi* populations collected from Shouguang on Shandong Province and from the Fangshan district of Beijing city in 2018

| Population | Collection location | Date | Latitude (°N), longitude (°E) |
| --- | --- | --- | --- |
| SGDY | Daotian town, Dongyi village | 20^th^ Nov. | 118.96, 36.86 |
| SGDL | Luocheng street, Dongliu village | 20^th^ Nov. | 118.91, 36.89 |
| SGZJ | Jitai town, Zhangjiamiaozi village | 20^th^ Nov. | 118.80, 36.86 |
| SGFQ | Luocheng street, Fuqiao village | 20^th^ Nov. | 118.92, 36.89 |
| SGL2 | Jitai town, Lvjia village | 20^th^ Nov. | 118.79, 36.78 |
| SGNC | Daotian town, Nanci village | 20^th^ Nov. | 119.00, 36.83 |
| SGL1 | Jitai town, Lvjia village | 11^th^ July | 118.79, 36.78 |
| BJFS | Fangshan district, Hongke farm | 9^th^ July | 115.86, 39.72 |

**Table S2** Linear model parameters estimated in the association between toxicity and concentration. *r*, correlation coefficient; X^2^, Chi-square value; *df*, degree of freedom.

| Population | Model parameters (estimate ± SE) | | | | |
| --- | --- | --- | --- | --- | --- |
|  | Slope ± SE | Intercept | *r* | X^2^ | *df* |
| SGDY | 1.14 ± 0.17 | 3.99 | 0.94 | 7.03 | 4 |
| SGDL | 1.26 ± 0.10 | 3.84 | 0.99 | 6.85 | 6 |
| SGZJ | 1.10 ± 0.10 | 3.95 | 0.98 | 6.49 | 5 |
| SGFQ | 1.14 ± 0.23 | 3.34 | 0.99 | 0.90 | 3 |
| SGL2 | 0.83 ± 0.10 | 3.79 | 0.94 | 9.92 | 4 |
| SGNC | 0.75 ± 0.11 | 3.85 | 0.97 | 3.57 | 5 |
| SGL1 | 1.25 ± 0.17 | 1.39 | 0.97 | 8.08 | 4 |
| SGL1-F5 | 2.52 ± 0.27 | 1.07 | 0.99 | 2.28 | 3 |
| BJFS | 1.43 ± 0.11 | 4.67 | 0.99 | 4.36 | 8 |
| BJFS-F5 | 1.89 ± 0.29 | 5.81 | 0.89 | 2.77 | 3 |

**Table S3** *P*‐values for tests of Hardy–Weinberg equilibrium in eight-greenhouse and two-laboratory populations of *Thrips palmi* based on microsatellite loci. Significant probabilities are in bold.

| Locus | SGDY | SGDL | SGZJ | SGFQ | SGL2 | SGNC | SGL1 | SGL1-F5 | BJFS | BJFS-F5 |
| --- | --- | --- | --- | --- | --- | --- | --- | --- | --- | --- |
| S02 | 0.6548 | 0.1144 | 0.5608 | 1.0000 | 1.0000 | 0.1124 | 0.5551 | **0.0000** | 0.6849 | 1.0000 |
| S03 | 0.7857 | 0.8429 | 0.3192 | 0.8692 | 0.5021 | 0.5852 | 0.2873 | 0.8935 | 0.1289 | 0.6059 |
| S04 | 0.6401 | 0.1284 | 0.1753 | 0.0203 | 0.2812 | 0.2379 | **0.0035** | **0.0071** | 0.5370 | 0.2080 |
| S06 | 1.0000 | 1.0000 | 0.0882 | 0.3679 | 1.0000 | 1.0000 | 0.1223 | 0.6827 | 0.1847 | 0.4644 |
| S09 | 0.0224 | 0.6128 | **0.0000** | 0.5716 | 0.2031 | 0.0141 | 0.2332 | 1.0000 | 0.5963 | 1.0000 |
| S11 | 1.0000 | 1.0000 | 0.0754 | 0.0889 | 1.0000 | 0.4959 | 0.0303 | 0.0874 | 0.6032 | 0.0585 |
| S12 | 0.2956 | 0.4142 | 0.0181 | 0.1441 | 0.6491 | 0.1636 | 1.0000 | 1.0000 | 0.2362 | 0.3657 |
| S17 | 0.6331 | 0.1136 | 0.7618 | 0.4674 | 1.0000 | 0.5063 | 0.6231 | 1.0000 | 0.4543 | 0.3326 |
| S19 | 0.3900 | 0.7779 | 0.3070 | 0.5156 | 0.7287 | 0.9549 | 0.6252 | 0.8291 | 0.0763 | 0.6456 |
| S22 | 0.0473 | 0.5727 | 0.4748 | 0.4473 | 0.9262 | 0.1353 | 1.0000 | 0.7482 | 0.5062 | 0.3703 |
| S23 | 0.2535 | 0.6475 | 0.8882 | 0.0353 | 0.6817 | 0.8163 | 0.6658 | **0.0016** | 0.1495 | 0.6512 |
| S25 | 0.2897 | 0.6044 | **0.0088** | 0.9646 | 0.9898 | 0.4177 | 0.1205 | 0.0360 | 0.6379 | 0.8304 |
| S27 | 0.8261 | 0.3464 | 0.2847 | 0.4096 | 0.8340 | 0.1661 | 0.6431 | 0.7717 | 0.8945 | 0.1359 |
| S29 | 0.6795 | 0.5304 | 0.8420 | 0.1098 | 0.7770 | 0.4205 | 0.1372 | 1.0000 | 0.3062 | 1.0000 |
| S31 | 1.0000 | 1.0000 | 0.4895 | 0.4805 | 1.0000 | 0.0706 | 0.7018 | 0.3862 | 0.9650 | 0.1885 |
| S32 | 0.3718 | 0.8161 | 0.6782 | 0.8653 | 0.2790 | 0.9238 | 0.9549 | 0.4664 | 0.5001 | 0.1647 |
| S36 | 0.3444 | 0.2880 | 0.2863 | 1.0000 | 0.5542 | 1.0000 | 1.0000 | 1.0000 | 1.0000 | 0.6220 |
| S40 | 0.7076 | 0.2094 | 0.9342 | 0.6384 | 0.0672 | 0.1227 | 0.5849 | 0.5195 | 0.9633 | 0.3669 |
| S42 | **0.0000** | 1.0000 | 0.5414 | 1.0000 | **0.0000** | 1.0000 | 0.3905 | 0.5569 | 0.2902 | 0.2333 |
| S51 | **0.0000** | 1.0000 | 0.1387 | 1.0000 | **0.0000** | **0.0000** | 1.0000 | 1.0000 | 0.1487 | 0.6521 |
| S56 | 0.6346 | 1.0000 | 1.0000 | 1.0000 | 0.1343 | 0.1124 | 0.2940 | **0.0024** | 1.0000 | 1.0000 |
| S57 | 0.9250 | 0.2442 | 0.3887 | 0.7125 | 0.2635 | 0.6541 | 0.8861 | 0.0295 | 0.3620 | 0.0707 |
